# MCL1 as putative target in pancreatoblastoma

**DOI:** 10.1007/s00428-022-03349-w

**Published:** 2022-06-07

**Authors:** Timm M. Reissig, Sebastian Uhrig, Philipp J. Jost, Claudio Luchini, Caterina Vicentini, Sven-Thorsten Liffers, Michael Allgäuer, Volkan Adsay, Aldo Scarpa, Rita Teresa Lawlor, Stefan Fröhling, Albrecht Stenzinger, Günter Klöppel, Hans-Ulrich Schildhaus, Jens T. Siveke

**Affiliations:** 1 Bridge Institute of Experimental Tumor Therapy, West German Cancer Center, University Hospital Essen, University Duisburg-Essen, Hufelandstrasse 55, 45147 Essen, Germany; 2 Division of Solid Tumor Translational Oncology, German Cancer Research Center (DKFZ) and German Cancer Consortium (DKTK), Partner Site University Hospital Essen, Heidelberg, Germany; 3 Department of Medical Oncology, West German Cancer Center, University Hospital Essen, Essen, Germany; 4 German Cancer Consortium (DKTK), Partner Site University Hospital Essen, Essen, Germany; 5 Computational Oncology Group, Molecular Diagnostics Program, NCT Heidelberg and DKFZ, Heidelberg, Germany; 6 Division of Applied Bioinformatics, German Cancer Research Center (DKFZ) and National Center for Tumor Diseases (NCT) Heidelberg, Heidelberg, Germany; 7 German Cancer Consortium (DKTK), Heidelberg, Germany; 8 Department of Medicine III, Klinikum rechts der Isar, TUM School of Medicine, Technical University of Munich, Munich, Germany; 9 Division of Clinical Oncology, Department of Medicine, Medical University of Graz, Graz, Austria; 10 Department of Diagnostics and Public Health, Section of Pathology, University and Hospital Trust of Verona, Verona, Italy; 11 ARC-NET Research Centre, University of Verona, Verona, Italy; 12 Institute of Pathology, University Hospital Heidelberg, Heidelberg, Germany; 13 Department of Pathology and Research Center for Translational Medicine (KUTTAM), Koç University, Istanbul, Turkey; 14 Division of Translational Medical Oncology, National Center for Tumor Diseases (NCT) Heidelberg and German Cancer Research Center (DKFZ), Heidelberg, Germany; 15 Department of Pathology, Technical University of Munich, Munich, Germany; 16 Institute of Pathology, West German Cancer Center, University Hospital Essen, University Duisburg-Essen, Hufelandstrasse 55, 45147 Essen, Germany

**Keywords:** MCL1, Amplification, FISH, Whole-genome sequencing, NCT MASTER, Pancreatoblastoma

## Abstract

Pancreatoblastoma (PB) is a rare tumor of the pancreas. In case of metastases, the treatment options are sparse and targeted approaches are not developed. We here evaluate *MCL1* amplification as a putative target in PB.

Thirteen samples from adult (10/13) and pediatric patients (3/13) were collected. Three of these samples had been previously subjected to whole-exome sequencing (2 cases) or whole-genome sequencing (1 case) within a precision oncology program (NCT/DKTK MASTER), and this analysis had shown copy number gains of *MCL1* gene. We established a fluorescence in situ hybridization (FISH) test to assess the copy number alterations of *MCL1* gene in 13 formalin-fixed paraffin-embedded PBs, including the 3 cases assessed by genome sequencing. FISH analysis showed the amplification of *MCL1* in 2 cases (both were adult PB), one of which was a case with the highest copy number gain at genomic analysis. In both cases, the average gene copy number per cell was ≥ 5.7 and the MCL1/1p12 ratio was ≥ 2.4. Our data support MCL1 as a putative target in PB. Patients with *MCL1*-amplified PB might benefit from MCL1 inhibition. Sequencing data is useful to screen for amplification; however, the established FISH for *MCL1* can help to determine the level and cellular heterogeneity of *MCL1* amplification more accurately.

## Introduction

Pancreatoblastoma (PB) is a very rare cancer but the most common malignant pancreatic tumor in young children [1]. It can also occur in adults at an even lower rate. Although PB seems to derive from the fetal anlage of pancreatic acinar cells and shows predominantly acinar differentiation, the exact origin of this tumor remains unclear [2]. Histologically, PB is a solid neoplasm with acinar differentiation and with the typical presence of squamoid nests, which represent its diagnostic hallmark. It is described as a slow-growing tumor [2] and the clinical presentation is unspecific with abdominal pain, mass effect, vomiting, and weight loss. When diagnosed, most tumors are rather large (> 5 cm) [1]. As a biomarker, alpha-fetoprotein (AFP) may be useful [3].

Complete surgical resection is the primary goal of the initial treatment which is feasible in the majority of cases [1]. Due to the high recurrence rate [4] and the fact that 17–35% of patients have metastases at the time of diagnosis [4, 5], (neoadjuvant) systemic chemotherapy is the therapy of choice. Most authors recommend cisplatin and doxorubicin (so-called PLADO schedule) based on anecdotal reference only given the rarity of the disease [1, 3].

Metastatic PB is associated with a dismal prognosis. Metastases primarily occur in the liver and less frequently in the lungs or regional lymph nodes [4]. The overall survival rates at 5 years for patients without metastases at presentation are 49% (95% confidence interval [CI], 31–66) and with metastases 25% (95% CI, 0–63). Post resection metastatic disease was associated with a significantly lower 5-year overall survival (21% [95% CI, 0–41] vs. 91% [95% CI, 83–100]). Collectively, these data demonstrate the need for additional therapeutic options. However, due to the rarity of this disease, clinical trials evaluating targeted therapies are neither feasible nor available.

Using next-generation sequencing (NGS) for metastatic PB from four adult patients (two samples were assessed by whole-exome sequencing, one sample by whole-genome sequencing, and one sample by targeted next-generation sequencing), Berger et al. identified molecular alterations that affected the FGFR signaling in three out of four patients which is potentially targetable and under current evaluation for different tumors [6]. The comprehensive sequencing identified a high-level amplification of *MCL1* encoding induced myeloid leukemia cell differentiation protein 1 (*MCL1*) in three patients. MCL1 belongs to the anti-apoptotic group of Bcl-2 (B-cell lymphoma 2) proteins regulating apoptosis. It has been well characterized in several tumor entities [7, 8], and MCL1 inhibitors have shown efficacy in preclinical trials for hematological neoplasms [9, 10] and solid tumors [11–13]. The prevalence of *MCL1* amplification in PB is unknown and might offer a rationale for targeted approaches with MCL1 inhibitors.

We here describe the analysis of *MCL1* amplification using FISH in 13 assessable cases to estimate the prevalence of *MCL1* amplification as potential biomarker in PB.

## Materials and methods

### Sample collection

This retrospective study was conducted on 13 formalin-fixed paraffin-embedded (FFPE) tumor samples from 13 patients with PB (Table 1). Three samples were obtained from the NCT/DKTK MASTER (Molecularly Aided Stratification for Tumor Eradication Research) cohort and previously published [6]. For whole-genome (patients 1 and 2), whole-exome (patient 3), or whole-transcriptome (patients 2 and 3) sequencing, fresh-frozen tissue specimens from the primary tumors of patients 1 and 3 as well as from a metastatic lesion of patient 2 were collected according to the standard protocols of the NCT/DKTK MASTER program [14, 15]. DNA extracted from buffy coats served as germline controls for the patients 3 and 2; a whole-blood sample was used for patient 1. Patients of the NCT/DKTK MASTER cohort gave written informed consent under protocol S-206/2011, which has been approved by the Ethics Committee of the University of Heidelberg. The present study was approved by the local Ethics Committee of the University Duisburg-Essen (20–9337-BO).

**Table 1:** Characteristics of cohort

Case #	Group	Age (years)	Gender	Total copy numbers (TCN)	Average gene copy number (avGCN)	MCL1/1p12 ratio	FISH results	Year of sample fixation
1	Adult	32	F	5.83	2.7	1.4	Negative	2013
2	Adult	18	M	6.72	**5.7**	**2.4**	**Positive**	2011
3	Adult	30	M	3.09	2	1.3	Negative	2016
4	Pediatric	3	F	n.a	2.0	0.9	Negative	1995
5	Pediatric	6	M	n.a	2.1	1.1	Negative	2003
6	Adult	55	M	n.a	2.8	0.9	Negative	2003
7	Adult	59	M	n.a	**6.7**	**2.6**	**Positive**	2005
8	Adult	32	W	n.a	2.3	1.2	Negative	2007
9	Adult	49	M	n.a	1.8	1.1	Negative	2008
10	Adult	63	F	n.a	1.6	1.3	Negative	2020
11	Adult	65	F	n.a	1.0	1.5	Negative	2013
12	Adult	69	M	n.a	1.0	1.6	Negative	2006
13	Pediatric	8	F	n.a	2.3	1.3	Negative	2018

*n.a.*, not available. Total copy numbers (TCN) were obtained from whole exome or whole genome sequencing. Average gene copy numbers (avGCN) and MCL1/1p12 ratios were obtained by fluorescence in situ hybridization (FISH)

### Next-generation sequencing and computational processing

The fresh-frozen tissue samples from patients 1 and 2 were subject to whole-exome sequencing (WES); the sample from patient 3 to whole-genome sequencing (WGS). In addition, we performed whole-transcriptome sequencing on the tumor/metastasis samples from patients 2 and 3. Library preparation, Illumina next-generation sequencing, and computational processing were carried out as described before [16].

### Fluorescence in situ hybridization (FISH)

FFPE samples were processed by using the ZytoLight FISH-Tissue Implementation Kit and SPEC MCL1/1p12 Dual Color Probe (Zytovision-Z-2173–200, ZytoVision GmbH, Bremerhaven, Germany). FISH assays were basically performed as previously described [17]. Pepsin digestion was used for proteolysis. For FISH evaluation, the entire tumor area was scanned for amplification hot spots. If MCL1 signals showed a homogenous distribution, random areas were used for reading the slides. Twenty contiguous tumor cell nuclei from three areas, either hot spots or from randomly selected regions, resulting in a total of 60 nuclei, were individually evaluated by counting green MCL1 and orange 1p12 signals. MCL1/1p12 ratio and the average *MCL1* copy number per cell were calculated and percentages of tumor cells with ≥ 4.0, ≥ 5.0, and ≥ 15.0 *MCL1* copies were recorded. All FISH assays were evaluated by one reader (HUS) and who was blinded to sequencing results. Based on the observed distribution of parameters within our cohort and comparison with sequencing data (see below), *MCL1* amplification was defined by a MCL1/1p12 ratio ≥ 2.0 and/or an average *MCL1* copy number per tumor cell ≥ 5.0.

## Results

### High-level amplification of MCL1 in pancreatoblastoma in adult patients

**Fig. 1:**
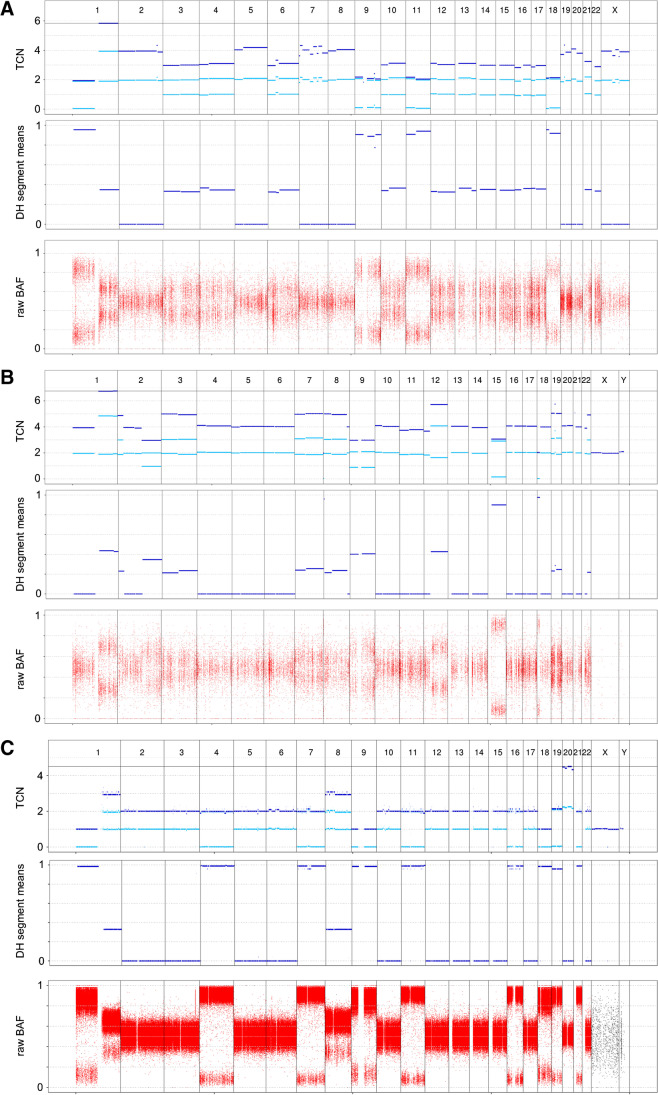
High total copy number (TCN) indicating MCL1 amplification. Total copy number (TCN), degree of homozygosity, and B-Allele Frequency (BAF) for the three adult patients with metastatic pancreatoblastoma in the MASTER cohort. Compared to the other chromosomes, chromosome 1q is highly amplified in all three patients with TCN from 3.09 up to 6.74

The NCT/DKTK MASTER (Molecularly Aided Stratification for Tumor Eradication Research), a multicenter, prospective observational study, analyzes tumors of advanced stage of young patients and rare tumors in search of potential therapeutic approaches [14, 15]. To date, four PBs were included and fresh-frozen tissue of three tumors was assessed by WES/WGS [6]. Analyzing all PB samples within NCT/DKTK MASTER for MCL1 alterations, copy number gains of *MCL1* were observed in all three PBs with total copy numbers (TCN) of 5.83, 6.74, and 3.09 for patients 1 to 3, respectively. The chromosomal region corresponding with MCL1, 1q21.2 [18], was one of the most amplified regions in patient 3 and the highest in patients 1 and 2 (see Fig. 1).

**Fig. 2:**
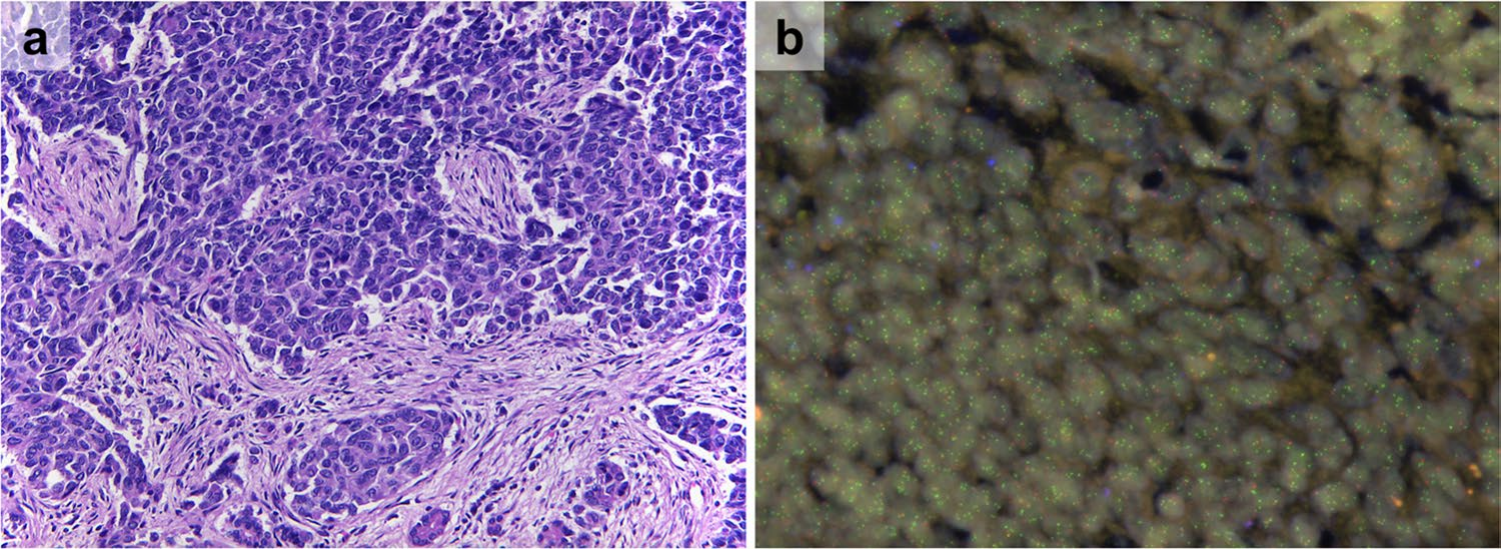
MCL1 FISH. **a** Pancreatoblastoma sample from an adult patient (H&E, original magnification: × 200). **b** MCL1 FISH. Orange signals label the reference locus on the short arm of chromosome 1 (1p12), green signals label a 575 kb chromosomal region on the long arm of chromosome 1 (1q21.3) including the MCL1 gene. This sample shows amplification of the MCL1 region. Non-neoplastic cells contain one or two orange or green signals

To further assess the amplification status of *MCL1* in PB, we collected twelve additional cases, totaling eleven adult and four pediatric cases. Patients’ characteristics and FISH results are summarized in Table 1. FISH was performed on 13 samples (10 adult, 3 pediatric cases). Two samples were found to be positive for *MCL1* amplification based on our established FISH criteria (see Fig. 2).

Assessing the NCT/DKTK MASTER samples, the sample with the highest TCN of 6.74 (patient 2) based on sequencing in the NCT/DKTK MASTER cohort also had a positive FISH result. MCL1/1p12 ratio was 2.5, average gene copy number was 5.7 per cell, and 80% of evaluated nuclei contained 5 or more MCL1 gene signals (93% ≥ 4.0 gene signals) (Fig. 1B). However, large signal clusters (≥ 15.0 gene signals per tumor cell) were not seen. Although the other two samples with sequencing data had TCN of 3.09 (patient 3) and 5.83 (patient 1), suggesting *MCL1* amplification, the FISH were negative. Percentages of tumor cells with ≥ 4.0 gene copies were 2% and 20%, respectively, but MCL1/1p12 ratio was < 2.0 and average gene copy number was < 3.0 in both samples.

Analyzing the other ten samples without sequencing data, another sample fulfilled FISH criteria for MCL1 amplification (patient 10) with a MCL1/1p12 ratio of 2.6 (average gene copy number: 6.7, tumor cells with ≥ 4.0, ≥ 5.0, and ≥ 15.0 gene signals were 88%, 82%, and 0%, respectively). Signal distribution showed moderate to marked heterogeneity among tumor samples and between tissue blocks.

Overall, 2 out of 10 (20%) samples of adult patients were positive for *MCL1* amplification, while no pediatric tumor showed amplification of *MCL1* with the caveat that only few pediatric cases were included in our cohort. We are not aware of any association of *MCL1* amplification with the clinical presentation including syndromal versus sporadic occurrence. Based on the NCT/DKTK MASTER inclusion criteria, patients with WES/WGS analyses were young adults (age range 18 to 50 years).

## Discussion

Metastatic PB lacks therapeutic options. Identifying genetic biomarkers might help to find novel targeted treatment strategies. Here, we assessed a series of 13 PBs for *MCL1* amplification by FISH after identification of amplified *MCL1* in cases from the NCT/DKTK MASTER cohort. We show that two out of 13 samples (15%) were FISH positive for *MCL1* amplification. Considering only samples from adult patients, the proportion of FISH positive cases increases to 20% (2/10). The exact frequency of *MCL1* amplification among various clinical settings of PB patients still needs to be prospectively determined.

Currently, chemotherapy is the main therapeutic option in metastatic PB. The most common agents include cisplatin and doxorubicin [1, 3] whereas FOLFOX/FOLFIRINOX (folinic acid, fluorouracil, oxaliplatin with or without irinotecan) is also administered to adult patients with metastatic disease [6]. Although targeted agents are lacking, reported therapeutic targets include the Wnt/ß-catenin pathway, IGF2, and the R-spondin/LGR5/RNF43 module [19]. Berger et al. detected FGFR alterations in three out of four patients and thus inhibition of FGFR signaling pathway might be a reasonable therapeutic approach with reported efficacy in some other cancer entities with FGFR aberrations [20–23].

MCL1 could be a candidate for targeted therapies currently evaluated in clinical trials (i.e., NCT04178902, NCT02992483). Preclinical evidence showed promising results in hematologic neoplasia such as acute myelogenous leukemia [9, 24] and multiple myeloma [24] as well as in some solid cancers [25]. In lung cancer, the combination of trametinib (MEK inhibitor) and AM-4907 (MCL1 inhibitor) showed tumor regression in xenograft tumors [11]. Adding AM-4907 to docetaxel or trastuzumab prolonged survival and induced tumor regression in two triple-negative and one HER2-amplified PDX breast cancer models [26]. Given our results of two identified cases with high-level *MCL1* amplification, MCL1 inhibitors might be a reasonable approach in *MCL1*-amplified PBs. Additionally, MCL1 inhibitors can be offered to patients where targeted therapies for the above-mentioned Wnt/ß-catenin pathway and R-spondin/LGR5/RNF43 module are lacking.

There remains uncertainty concerning the true proportion of PBs with *MCL1* amplification, although our cohort comprising 13 analyzable patient samples can be regarded as one of the larger series for this very rare tumor. The rarity of PB renders comprehensive analyses in large cohorts difficult. Bigger consortia on rare cancers such as the EXPeRT [1] or the Italian TREP project [27] might help to determine the frequency. As most biomaterial in our sample set was considerably older, only basic clinical parameters could be retrieved.

FISH is an established and straight-forward applicable technique in clinical routine diagnostics for the detection of amplifications with the potential of prognostic or predictive biomarkers in various cancer entities [28–32], e.g., ERBB2 in breast and gastric cancer [33–35]. Criteria for amplification obtained by FISH need to be carefully established and are dependent on and specific for (i) the gene of interest and (ii) the tumor subtype. One approach to establish FISH positivity criteria is to analyze larger cohorts of a tumor entity and to describe the unbiased distribution of parameters. Thus, unequivocal amplification levels can be defined [28]. In this study, a larger cohort of PB samples was investigated by MCL1 FISH. Based on our observations, criteria for FISH positivity in PB could be defined (*MCL1* amplification: MCL1/1p12 ratio ≥ 2.0 and/or average gene MCL1 count per tumor cell ≥ 5.0). By applying these criteria, we could identify two *MCL1*-amplified cases among ten evaluable PB samples from adult patients. One of these two FISH-positive samples was also sequenced in the NCT/DKTK MASTER cohort and had the highest total copy number for MCL1 of all sequenced PB samples. Although sequencing data showed higher TCN for MCL1 in the other two samples from the NCT/DKTK MASTER cohort, the MCL1 FISH did not confirm these results. First, lower values of TCN in patients 1 and 3 might be a reason. Second, technical issues might interfere with the analyses as fresh frozen tissue has to be strictly kept at less than − 80 °C and even short periods of more than − 80 °C might lead to degradation of the tissue. Moreover, another bias might come from the different tissues analyzed as the NGS data were acquired from fresh frozen tissue whereas the FISH analyses were performed on FFPE tissue. Third, other contributing factors may include sensitivity differences, clonal heterogeneity, sampling bias, and different tumoral ploidy. Intratumoral heterogeneity has been demonstrated in PB as well, and genetic heterogeneity seemed to be associated with morphologic differentiation lineages in a reported case of a PB patient [36]. We observed MCL1 amplification in cellular areas with more basophilic appearance. Further evaluations of larger case series, however, are needed to clarify potential associations between differentiation lineages and MCL1 amplification.

Based on our findings, we suggest utilizing WES/WGS or FISH for detecting *MCL1* high level amplification in PBs to identify patients as potential candidates for a clinical trial or individual personalized treatment with MCL1 inhibitors. Also, other NGS applications including hybrid capture and amplicon-based NGS may be used if carefully established and validated on FFPE materials. If FISH is applied, we propose a potential definition for assay positivity. As a caveat, the predictive value of our approach remains to be validated with clinical treatment data. FISH as a technology has some clinical advantages since it is fast and works usually reliably with FFPE material even if tissue blocks contain only few tumor cells. Intratumoral heterogeneity was observed, which requires careful screening of tumor samples for amplification hotspots. Thus, we regard our definition of MCL1 FISH positivity as preliminary and a subject to potential adjustments. However, NGS-based findings should be validated by FISH analysis. This is why we suggest MCL1/1p12 ratio ≥ 2.0 and/or average MCL1 gene count per tumor cell ≥ 5.0 as reasonable selection criteria for potential targeted treatments since these criteria reflect the highest unequivocal amplification level based on our data from a larger series of adult and pediatric PBs. In contrast to sequencing methods, especially comprehensive ones such as whole-genome or whole-exome sequencing, FISH is a robust, cheap, fast, and easily applicable method which may be useful to identify patients potentially benefitting from targeted therapy.

In summary, FISH criteria were established and *MCL1* amplification was identified in a subset of adult patients with PB. Given available MCL1 inhibitors, our study supports the rationale to test MCL1 amplification in a clinical setting to evaluate targeted treatment approaches.

## Data Availability

The data that support the findings of this study are available from the corresponding authors upon reasonable request.
